# The Effect of Sitz Bath of Hydro-Alcoholic Extract of Myrrh Gum on Episiotomy Wound Healing in Nulliparous Women 

**Published:** 2019-06

**Authors:** Zahra Sarbaz, Zahra Yazdanpanahi, Ayda Hosseinkhani, Farzaneh Nazari, Marzieh Akbarzadeh

**Affiliations:** 1 Department of Midwifery, Community Based Psychiatric Care Research Center, School of Nursing and Midwifery, Shiraz University of Medical Sciences, Shiraz, Iran; 2 Department of Pharmacology, Research Center for Traditional Medicine and History of Medicine, Shiraz University of Medical Sciences, Shiraz, Iran; 3 Department of Obstetrics, School of Medicine, Bushehr University of Medical Sciences, Bushehr, Iran; 4 Department of Midwifery, Maternal –fetal Medicine Research Center, School of Nursing and Midwifery, Shiraz University of Medical Sciences, Shiraz, Iran

**Keywords:** Episiotomy, Wound Healing, Nulliparous Women, Myrrh Gum

## Abstract

**Objective:** To investigate the effect of sitz bath of hydro-alcohol extract of myrrh plant on episiotomy wound healing.

**Materials and methods:** the clinical trial was performed on 60 nulliparous women from July 2017 to December 2017. After episiotomy, the intervention and control groups respectively underwent sitz bath of myrrh gum and normal saline for 7 days. Data collection was REEDA scale.

**Results:** Significant difference between the mean score of redness, proximity of wound edges to each other and REEDA scale in both groups on the third, seventh and tenth days after delivery (p < 0.05). There was a significant difference between mean scores of bruise on the 10^th^ day and wound discharge on the 7^th^ and 10^th^ days. The mean scores of bruise were not significantly different between the two groups (p > 0.05).

**Conclusion:** The effect of this myrrh plant on episiotomy wound healing is greater and faster than the effect of usual cares.

## Introduction

Episiotomy incision is almost common for successful vaginal birth in all primiparouse (1). The usual use of episiotomy has decreased in advanced countries, but women in Asian countries are prone to wide tear of perineum due to short perineum and tight tissue; therefore, this method is commonly used in these countries (2). Delay in wound healing increases the risk of infection and unpleasant anatomical outcomes, and the infection can lead to dangerous complications and even death (3). Observing perineal hygiene, drying the wound site, and using different treatments both in pharmaceutical and non-pharmaceutical methods are among the measures that can be done after delivery to accelerate wound healing. In choosing pharmaceutical therapies, there should be some considerations despite clinical trials and empirical documentations for using them. Digestive problems and dangers caused by medicine transfer through breastfeeding and possible complications for the child are among these considerations (4). 

Myrrh is one of the plants with the chemical composition and its main components are resin gum, 2.5-5% essential oil including terpene, 30-40% gum containing arabinose, galactose, and methyl GoliCoronkyacid; the remainder is about 30-40% resin. About 60% of the resins are alcohols and terpenic acids (5), and since research on postpartum maternal health problems is limited and inadequate in the world, the World Health Organization has announced the necessity of research in this regard as a priority (6). On the other hand, according to the evidence in Iranian traditional medicine and studies conducted on myrrh plant, it seems that this plant can be useful in episiotomy wound healing. Since the search in databases revealed that there was no study in this regard using this method, the information gap is quite tangible in this regard. Therefore, this research aimed to investigate the impact of sitz bath of hydro-alcohol extract of myrrh gum plant on episiotomy wound healing.

## Materials and methods

**Figure1:**
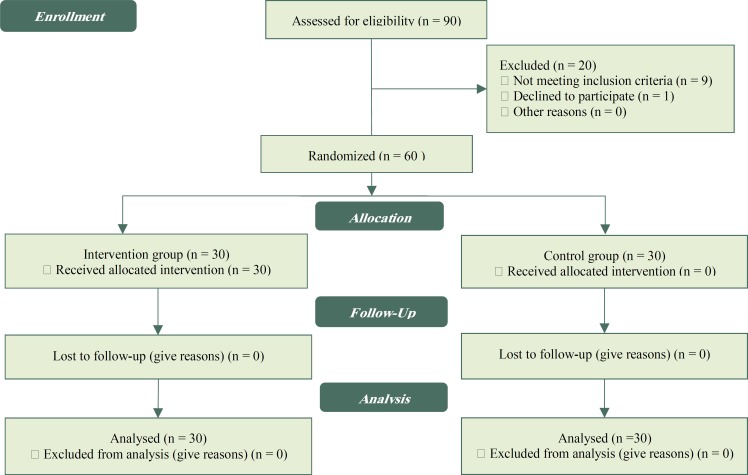
flow diagram for the study

This double blind clinical trial was performed on 60 nulliparous women referred to Salman Farsi Hospital of Bushehr from July 2017 to December 2017. Since there was no similar research regarding the use of myrrh plant and its effect on wound healing using REEDA scale, the results obtained from two groups of ten patients with and without the use of myrrh plant were used asthe pilot to determine the sample size. The sample size was calculated 24 in each of the two groups, which increased to 30 by considering 20% reduction in the sample size. In total, sample size was obtained 60. Individuals were selected based on convenience sampling and considering inclusion criteria. The subjects were divided into two groups using permuted block randomization, and 30 blocks of 2 subjects were selected (Figure 1). 

Inclusion criteria included the following features: nulliparous women, gestational age of 37-42 complete weeks, vaginal delivery with medio-lateral episiotomy, singleton birth, cephalic presentation, pre-pregnancy body mass index less than 25, no experience of surgery on the vagina and perineum, having no specific disease, no three and four grade rupture, or rupture other than episiotomy, no smoking during pregnancy, lack of varicose veins in the perineum, lack of genital warts and sexually transmitted infections, big baby and dystocia, lack of intercourse, and heavy labor. 

Manual removal of the placenta, postpartum abnormal vaginal bleeding, hematoma, postpartum fever and chills, perineal manipulation after delivery, episiotomy site infection.

A usable solution of hydro-alcohol extract of myrrh gum was prepared and coded in the form of 1:5 tincture of myrrh gum in 90% alcohol in the aseptic condition and under the control of a pharmacologist at the Faculty of Pharmacy in Shiraz University of Medical Sciences. The placebo was also prepared under the control of a pharmacologist at the Faculty of Pharmacy in Shiraz University of Medical Sciences in glass bottles with the same form and color. It was then coded by the pharmacist’s professor without informing the researcher.

In order to control the interventional factors in the delivery room, the researcher presented the patient and investigated how episiotomy was done and how it was repaired by the midwife through direct observation. After washing the episiotomy site with water, the researcher dissolved 10cc of alcoholic extract in 5 liters of water for the first time, and the mother used the sitz bath of this solution for 10 minutes; she was using this sitz bath twice a day in 7 days after delivery. 140cc of the aforementioned tincture coded in dark color 10 cc bottles was given to the mother. For all groups, routine hospital cares were explained with respect to ethical considerations. After mother’s delivery, the researcher attended the ward and carried out an initial assessment of the episiotomy healing score using REEDA scale, before the intervention (the first 4 hours after episiotomy) when the effect of lidocaine was dissipated. Then, he performed the intervention at 4 o’clock after episiotomy; the routine cares, how tincture is used, and time of the next reference was taught to mothers through an educational pamphlet. Since this was a blind study, coded cards were given to the mothers. They were asked to contact the researcher if they had any problems or questions. 

The healing rate of episiotomy wound was investigated by the researcher at the first four hours after delivery and before the intervention on the third, seventh and tenth days after delivery, using REEDA scale at the Midwifery Center of Salman Farsi Hospital. The REEDA scale is a tool designed by Davidson in 1974 to examine the perineum in terms of redness, edema, bruising, discharge and proximity of wound edges to each other. According to table 1, a score of 0 to 3 was considered for each variable. The scores obtained from each variable were summed, and the summed score ranged from 0 to 15, indicating the degree of wound healing. The closer this scores to zero, the better episiotomy repair. Reliability and validity of this research tool have been confirmed in various studies. In Iran, reliability and validity of REEDA scale were confirmed by Pazandeh. To assess its reliability, the method of measuring among the observers was used (7). The data collection method was based on examination and direct observation. Data were collected using a researcher-made questionnaire including individual characteristics (age, body mass index, education, employment status, economic status) and midwifery-neonatal data form (length of episiotomy, number of surface sutures, length of delivery stages, baby’s head circumference), and a checklist for evaluating the condition of wound healing, in which the rate of episiotomy wound healing was evaluated using REEDA scale (Table 1).

**Table 1:** Evaluation of episiotomy status based on REEDA table

	**Points**			
	**0**	**1**	**2**	**3**
Red	Does not have	0.25 centimeters from each side	0.5 centimeters from each side	More than 0.5 centimeters across each side
Sowllen	Does not have	In the perineum, less than 1 cm from the cut area	Between 1-2 cm from the cut in the perineum and vulva	In perineal and volvo more than 2 centimeters of cut
Bruise	Does not have	About 0.25 centimeters on either side of the cut or 0.5 centimeters at one end of the cut	About 1 cm on either side of the cut, or 2-5 / 0 cm on one side of the cut	More than 1 cm on either side of the cut or more than 2 cm on one side
Secretion of the wound	Does not have	Serozo	Watery-bloody	Bloody – purulent
Two-edge wound spacing	Closed	Separation of 3 mm or less double-edged skin	Separation of the skin and subcutaneous fat layer between the wound edges	Separation of the subcutaneous fat and the facial layer of the two- edges of the wound

***Statistical analysis:*** In order to achieve the research goals, the demographic characteristics of the mothers were first studied, considered in two groups, and compared. The main variables of the two groups were then compared using chi-square test, Fisher, ANOVA, Friedman, independent t-test and paired t-test. Data were analyzed using SPSS- 21, and the significance level was considered 0.05.

***Ethical consideration:*** This research project was approved by the local Ethics Committee of Shiraz University of Medical Sciences and written informed consents were obtained from all the participants. The research proposal No. 12736 financially supported by endocrine and metabolism research center, Shiraz University of Medical Sciences. The research in Iranian Registry of clinical trial has registered with registration number: IRCT2016120331219N1.

## Results

The highest percentage of the units under investigation in the control group and group of myrrh plant was 63.3% in the age group of 21-30 (Table 2). 

**Table 2:** Individual characteristics of the research units

**Variable**		**Case** **group**	**Control** **Group**
		**Number ** **(percent)**	**Number ** **(percent)**
Age	20>	6(20)	7(23.3)
	30-21	19(63.3)	19(63.3)
	31 <	5(16.7)	4(13.3)
BMI	19.8 >	2(6.7)	5(16.7)
	19.9-26	28(93.3)	25(83.3)
Level of Education	Under the diploma	1(3.3)	3(10)
	Diploma	15(50)	12 (40)
	Academic	14(46.7)	15(50)
Job	Employed	2(6.7)	2(6.7)
	Housewife	28(93.3)	28(93.3)
The economic sitiuation	Weak	1(3.3)	1(3.3)
	Medium	27(90.0)	27(90.0)
	Top	2(6.7)	2(6.7)

Half of the samples under investigation in the myrrh plant group and 66.6% in the control group had an episiotomy length of 4cm. The results obtained from ANOVA test showed that there was no significant difference between the number of surface sutures in both groups (p = 0.24).The results of paired t-test showed that there was no significant difference among the length of labor stages (first stage (p = 0.41), second stage (p = 0.331), and third stage (p = 288)) between the two groups. The results of ANOVA test showed that there was no significant difference between the two groups in terms of the baby’s head circumference (p = 0.064). Regarding midwifery-neonatal characteristics, the results of statistical tests showed that there was no significant difference between the two groups (Table 3).

**Table 3:** Midwifery -infant profile of the units under study

**Variable**	**Category**	**Case** **Group**	**Control** **Group**
		**Number ** **(percent)**	**Number ** **(percent)**
Episiotomy cutting length	2	1(3.3)	0(0)
	3	12(0)	5(16.7)
	4	15(50)	20(66.6)
	5	1(3.3)	5(16.7)
	6	1(3.3)	0(0)
Number of surface sutures	4	6(20)	6(20)
	5	8(26.7)	12(40)
	6	13(3.43)	7(23.3)
	7	2(6.7)	2(6.7)
	>8	1(3.3)	3(10)
Length of delivery stages	Stage one	190.50	157.50
	Stage two	46.50	55.17
	stage three	7.50	8.67
Round the head of the baby	30	2(6.7)	2(6.7)
	32	3(10.0)	3(10.0)
	33	1(3.3)	5(16.6)
	34	20(46.7)	17(56.7)
	35	3(10.0)	2(6.7)
	36	1(3.3)	1(3.3)

Based on investigating episiotomy in terms of redness of the wound site on the third day (p = 0.003), seventh day (p = 0.001), and tenth day (p = 0.001), there was a significant difference between the scores of the two groups. Also, based on investigating the score of edema, there was no significant difference between the two groups before the intervention (p = 0.923), and on the third day (p = 0.236), seventh day (p=0.241), and tenth day (p = 0.331) after delivery. There was a significant difference between the two groups in terms of investigating bruising variable on the 10^th^ day after delivery (p = 0.041).

Also, in terms of wound discharge on the seventh day (p = 0.014) and tenth day (p = 0.001) after delivery, there was a significant difference between the two groups. 

**Table 4:** Comparison of two groups using Kruskal-Wallis intergroup test

**Variables**	**Category**	**Before intervention**	**Third day**	**Seventh day**	**Tenth day**
		**Mean (SD)**	**Mean (SD)**	**Mean (SD)**	**Mean (SD)**
Red	Case	0.13(0.346)	0.13(0.346)	0.37(0.490)	0.37(0.490)
	Control	0.3(0.183)	0.53(0.571)	0.87(0.571)	0.90(0.607)
	P- value	0.988	0.003	0.001	0.001
Sowllen	Case	0.03(0.183)	0.07(0.254)	0.07(0.254)	0.10(0.305)
	Control	0.03(0.183)	0.17(0.379)	0.23(0.430)	0.27(0.450)
	P- value	0.923	0.236	0.241	0.331
Bruise	Case	0.07(0/254)	0.07(0.254)	0.03(0.180)	0.03(0.180)
	Control	0.00(0.000)	0.10(0.305)	0.17(0.379)	0.20(0.407)
	P- value	0.155	0.647	0.219	0.041
Secretion of the wound	Case	0.00(0.000)	0.23(0.430)	0.50(0.509)	0.57(0.567)
	Control	0.00(0.000)	0.47(0.507)	1.17(0.531)	1.23(0.568)
	P- value	0.999	0.060	0.014	0.001

Based on investigating the proximity of wound edges to each other on the third day (p = 0.001), seventh day (p = 0.001), and tenth day (p = 0.019) after delivery, there was a significant difference between the two groups. Comparing the total scores of REEDA scale on the third day (p = 0.010), seventh day (p = 0.000), and tenth day (p = 0.000) after delivery showed that there was a significant difference between the two groups (Table 4).

As to the redness variable between the periods before and after the intervention in each group, it was shown that there was a significant difference between the two intervention and control groups (p < 0.001). It means that redness was observed in both groups, but it was less in the myrrh plant group. Also, the investigations of variables of inflammation (p < 0001), bruising (p < 0001), discharge (p < 0001), proximity of wound edges to each other (p < 0001), and total scores of REEDA scale in each group showed that there was a significant different between the two groups (p < 0001) (Table 5).

## Discussion

Episiotomy has physiological, psychological, economic and social effects on women. How to do this technique and the quality of future cares is of particular importance (8), and delay in wound healing increases the risk of infection and unpleasant anatomical outcomes; also, infection can lead to dangerous complications and even death.

This research compared the effect of sitz bath of hydro-alcoholic extract of myrrh gum and placebo. A to the REEDA tool, it was shown that the myrrh plant group had a greater effect on the reduction of redness mean score than the control group. In the study conducted by ArdeshiriLagimi et al. (2009), it was concluded that herbal therapy had a stimulating effect on the growth of the fibroblastic cells, and it reduced redness in episiotomy (9). 

In Albishiri’s prospective study was showed that myrrh may be effective in improving mouth sores in patients with Behcet’s disease (10).

**Table 5:** Comparison of two groups using Friedman's intra-group test

**Variable**	**Category**	**Before intervention**	**Third day**	**Seventh day**	**Tenth day**	**P- value**
		**Mean (SD)**	**Mean (SD)**	**Mean (SD)**	**Mean (SD)**	
Red	Case	0.13(0.346)	0.13(0.346)	0.37(0.490)	0.37(0.490)	0.057
	Control	0.3(0.183)	0.53(0.571)	0.87(0.571)	0.90(0.607)	0.000
Sowllen	Case	0.03(0.183)	0.07(0.254)	0.07(0.254)	0.10(0.305)	0.000
	Control	0.03(0.183)	0.17(0.379)	0.23(0.430)	0.27(0.450)	0.063
Bruise	Case	0.07(0.254)	0.07(0.254)	0.03(0.180)	0.03(0.180)	0.000
	Control	0.00(0.000)	0.10(0.305)	0.17(0.379)	0.20(0.407)	0.000
Secretion of the wound	Case	0.00(0.000)	0.23(0.430)	0.50(0.509)	0.57(0.567)	0.000
	Control	0.00(0.000)	0.47(0.507)	1.17(0.531)	1.23(0.568)	0.000
Two-edge wound spacing	Case	0.00(0.000)	0.00(0.000)	0.23(0.430)	0.40(0.563)	0.000
	Control	0.00(0.000)	0.30(0.466)	0.77(0.679)	0.80(0.714)	0.000
Total score	Case	0.10(0.403)	0.50(0.777)	1.23(1.031)	1.43(1.305)	0.000
	Control	0.17(0.461)	1.30(1.466)	1.28(3.07)	3.23(1.406)	0.000

The results of this investigation are consistent with those of the present study, and the evidence of wound healing was observed in both studies.

In the study conducted by Abbasizadeh et al., Stevenson et al. (2002) in London and Al-Zahrani et al.), it was found that a herbal complex containing myrrh alone or with other plants is effective on diabetic wound and redness of the wound area (11-13). Since inflammation is the body’s defensive response against damage or tissue damage, pain, warmth, redness, swelling and functional decline are localized inflammatory symptoms (14). Therefore, it can be suggested that the myrrh plant has a faster effect in reducing the mean score of redness than routine treatment; probably, it is due to furanoeudesma-1, 3-diene in this plant which has a strong anti-inflammatory effect. The results of the present research is in the same line with those of these studies; however, the present research is the first study investigating the effect of myrrh plant on episiotomy redness. Comparison of the mean score of inflammation before the intervention and on days3, 7 and 10 after delivery revealed that there was no significant difference between the two groups of intervention and control. In the study conducted by Vakilian (2007), it was found that the mean score of episiotomy wound redness in the lavender group on the fifth day was less than the one in the betadine group, but edema was significantly higher (15). The present research was consistent with the above-mentioned study, and edema increased during 10 days with the difference that in the above-mentioned study, edema in the lavender group on the fifth day was more than that in the control group. However, in this study, despite the increase in edema in the myrrh group on the tenth day, edema was less than that in the control group. The similarity of these two studies may be due to the similarity in the position of individuals and how the herbal medicine was used. This research is not consistent with the study conducted by Hoffer in 2013, which aimed to investigate the effect of myrrh plant on blood leukocytes, peptic ulcer disease and skin ulcers on the face. Since the WBC level has been reduced after treatment in the skin-damages group treated by myrrh plant and peptic ulcer disease group treated by myrrh plant compared to before injury, it was concluded that myrrh probably contains a substance that can show anobvious antigenic response. Also, it helps maintain high level of WBC during the treatment (16). Consequently, myrrh probably contains a substance that can show an obvious antigenic response, and it has anti-inflammatory properties and can reduce the edema that was not observed in the present study; perhaps, it was because of the difference in the amount, concentration, the method of using myrrh, and the difference in the human and animal samples. The present research is not consistent with the study conducted by Al-Mobeeriek in 2014, which aimed to investigate the effect of myrrh on aphthous ulcer compared to treatment by tetracycline and chlorhexidine gluconate. 

In the study conducted by Al-Mobeeriek, it was found that treatment with myrrh improved and repaired the damaged tissues in a short period of time after using it (less than 2 weeks), and the result was reversed in long periods. (17). Perhaps, the inconsistency of these studies with the present research is due to the extent and intervals and method of using myrrh plant. Comparison of the mean score of bruising showed that there was a significant difference between the two groups in terms of the mean score of bruising 10 days after delivery. There was no significant difference between the two groups before the intervention and 3 and 7 days after delivery. The results of the study conducted by Impellizzeri et al. (2011) showed that herbal therapy could help improve bruising (18). In the study carried out by Sharifi et al. in 2014-2015, which aimed to investigate the effect of Scophulariastriataon episiotomy wound healing, it was concluded that there was a significant difference between the two groups on the fifth, tenth and twenty-first days after delivery, in terms of mean scores of variables such as redness and edema and total score, and there was no significant difference between the mean scores in the two groups during the investigation (19). Pharmacological and toxicological studies on various species of Commiphora plant showed that this plant had anti-inflammatory, anti-parasitic, antimicrobial, antioxidant, anti-diabetic and hepatic and heart protective properties, etc. (20). In the study conducted by Abedi et al. (2017), it was concluded that Commiphora plant had suitable and acceptable healing effects on the repair of the sciatic nerve damage in rabbits (14). Comparison of the mean score of discharge variable showed that there was a significant difference between the two groups in terms of the mean score of episiotomy site discharge 7 and 10 days after delivery (p < 0.05). The results showed that over time, myrrh plant had more positive effects on discharges as compared to the control group. In a prospective study carried out by Ghannam et al. (2016) during 8 years entitled myrrh causing surgical site infection, it was indicated that wound infections were found in 15 patients (over 90% of patients had used myrrh on their wounds). It was concluded that myrrh extract led to surgical site infection (SSI); it was applied on wounds after surgery, and it should be avoided (21).

The result of this study is not consistent with that of the present research; this may be due to the difference in the method of study, type of ulcers and quality of caredelivered after surgery. In the study carried out by Yadeghar et al. (2013) on tissue pathology, healing rate was significantly better in the group receiving Commiphoramyrrha extract, and Commiphoramyrrha extract had a positive effect on burn treatment, and had more appropriate healing effect in a concentration of 2.5% compared to silver sulfadiazine (22). This is consistent with the findings of the present research, and myrrh plant was used in both studies for wound healing; it had better effects on wound healing, which may be due to the fact that myrrh plant has antibacterial, antiviral and antifungal effects leading to dryness of the wound discharges (23). Also, this plant is effective on skin pathogenic microbes, including Candida and Staphylococcus aureus (24). Furanoeudesma-1, 3-diene in this plant has antibacterial and antiviral effects (25); therefore, it can be concluded that myrrh plant does not cause wound infections and episiotomy wound discharge. Comparison of the mean scores of proximity of wound edges to each other showed that wound dehiscence in myrrh plant group was less than that in control group, and it accelerated wound healing. The mean score of proximity of episiotomy wound edges to each other on the tenth day after delivery in the control group was twice that in the myrrh group. Also, myrrh plant had a greater impact on the proximity of wound edges than usual cares. The study by Behmanesh et al. (2012), which aimed to investigate the effect of sitz bath of olive oil on the improvement of perineal damage after delivery, showed that there was a significant difference between the two groups in, redness on the fifth day and redness and edema on the tenth day after delivery. But these cases were found in the control group; the difference was not significant. The difference observed in the olive oil group was positive within the first and tenth days; it means that REEDA scale on the tenth day was less than the score on the first day (7). The result of his study is consistent with that of the present research, with the difference that REEDA scale increased in both groups in the present research, and RREDA score in the control group was approximately three times that of the myrrh group on the tenth day. 

Myrrh plant can improve episiotomy wound, which can be due to its effect on stimulating granulation and increasing glycoproteins and collagen in the wound. The effects of myrrh plant on wound healing have been shown in other studies, including the study by Abbasizadeh et al. (2015) in which myrrh plant was shown to improve the surgical wound by promoting epithelization (11). Sue et al. (2010) showed that various Commiphoramyrrha extracts had anti-inflammatory, antimicrobial and analgesic effects (26). Also, Helal et al. (2005) reported the effect of its extract on reducing blood glucose by increasing insulin and restoring the pancreas beta cells and increasing the serum insulin levels (27). 

The comparison of total mean score of the scale showed that reduction of REEDA scale in the myrrh plant group was more than that of the control group. Therefore, it can be said that the effect of myrrh plant on reducing REEDA scale is higher than that in the control group, and it shows faster and higher rate of reduction than the control group. Comparison of REEDA scale in this study showed that the mean score of episiotomy wound healing in the myrrh plant group three days after delivery was almost half of that in the control group, and the mean score in the control group was three times that of the myrrh group on the tenth day; this indicates that wound healing rate was higher in the group using myrrh plant. Studies have shown that Commiphora myrrha can prevent the activity of cancer cells and is effective on eight lines of cancer cells as well as normal human mammary epithelial cells. Since myrrh plant contains flavonoids with antioxidant properties, myrrh is probably effective on episiotomy wound healing by neutralizing oxygen free radicals. 

***Limitations of the study:*** Different delivery agents from the beginning of sampling to the end of the process.

    -   The correct use of hydro-alcoholic extract or observance of its application time which were controlled by face-to-face tutorials and giving the researcher’s contact number to the samples.

    -   Delay or non-reference of subjects at the time specified, which was controlled by giving coded cards, determining the data of visit and the researcher’s contact with subjects.

    -   Control and observance of health conditions, number of meals or nutrient groups related to research samples were among research limitations that were somewhat controlled by postpartum education and counseling.

## Conclusion

The hydro-alcoholic extract of myrrh plant is effective on accelerating the episiotomy wound healing process. Therefore, due to the beneficial effects and fewer side effects of hydro-alcoholic extract of myrrh plant on episiotomy wound healing in women, it is suggested that myrrh can be used as an effective and appropriate method with minimal side effects at the hospital and home in order to have faster episiotomy wound healing. 

Based on the results of this study, it is recommended that the effect of this myrrh plant on episiotomy wound healing is greater and faster than the effect of usual cares. This can be due to curative, anti-inflammatory and antimicrobial properties of myrrh plant. Also, it seems that more clinical trials are needed to evaluate the effect of this plant on wound healing by using different doses of the drug.
